# Computational Pathology Detection of Hypoxia-Induced Morphologic Changes in Breast Cancer

**DOI:** 10.1016/j.ajpath.2024.10.023

**Published:** 2024-12-26

**Authors:** Petru Manescu, Joseph Geradts, Delmiro Fernandez-Reyes

**Affiliations:** ∗ Department of Computer Science, Faculty of Engineering Sciences, University College London, London, United Kingdom; † Department of Pathology, University of California San Francisco, San Francisco, California

## Abstract

Understanding the tumor hypoxic microenvironment is crucial for grasping tumor biology, clinical progression, and treatment responses. This study presents a novel application of artificial intelligence in computational histopathology to evaluate hypoxia in breast cancer. Weakly supervised deep learning models can accurately detect morphologic changes associated with hypoxia in routine hematoxylin and eosin (H&E)–stained whole slide images (WSIs). The HypOxNet model was trained on H&E-stained WSIs from breast cancer primary sites (*n* = 1016) at ×40 magnification using data from The Cancer Genome Atlas. Hypoxia Buffa signature was used to measure hypoxia scores, which ranged from −43 to 47, and stratified the samples into hypoxic and normoxic based on these scores. This stratification represented the weak labels associated with each WSI. HypOxNet achieved an average area under the curve of 0.82 on test sets, identifying significant differences in cell morphology between hypoxic and normoxic tissue regions. Importantly, once trained, the HypOxNet model required only the readily available H&E-stained slides, making it especially valuable in low-resource settings where additional gene expression assays are not available. These artificial intelligence–based hypoxia detection models can potentially be extended to other tumor types and seamlessly integrated into pathology workflows, offering a fast, cost-effective alternative to molecular testing.

## Graphical Abstract

**Figure:**
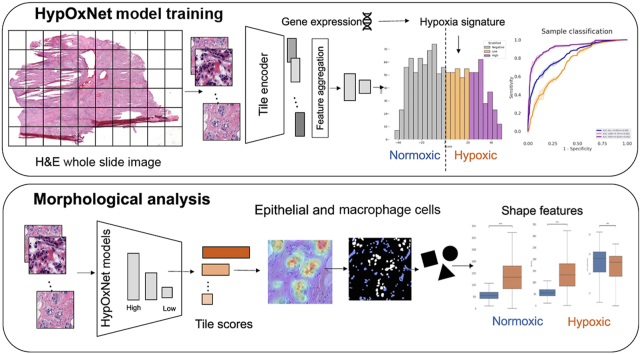

During the growth of malignant tumors, the neoplastic cells can outgrow their blood supply, leading to an insufficient delivery of oxygen and nutrients. As a result, regions within the tumor experience a shortage of oxygen, generating a hypoxic^1^ microenvironment, which is a hallmark of solid tumors, including breast cancer.^2^, ^3^, ^4^ Hypoxia can induce genetic instability in tumor cells, leading to the accumulation of genetic mutations and potentially driving tumor progression and resistance to treatment. For instance, hypoxic tumor cells are more resistant to radiation therapy compared with well-oxygenated cells because of their decreased sensitivity to oxidative stress.^5^ Adjuvant radiation therapy following breast-conserving surgery is part of standard treatment for early-stage breast cancer, as it considerably decreases risk for ipsilateral breast tumor recurrence.^2^ However, hypoxic regions within residual tumor foci may not receive an effective dose of radiation during treatment, leading to an increased risk of recurrence.^2^^,^^6^ Therefore, detecting hypoxia in solid primary tumors is important to identify potential patient groups who do or do not benefit from certain therapies.^5^ Moreover, hypoxia-induced changes in gene expression can facilitate the metastatic process, increasing the likelihood of cancer spreading to other parts of the body.^1^

Hypoxia can be quantified on the basis of the expression patterns of specific genes known to be regulated by hypoxia, such as those under the control of the hypoxia-inducible factor-1. The expression levels of these hypoxia-regulated genes collectively indicate the degree of hypoxia in the tumor. Microarray- or RNA-sequencing technologies are usually used to analyze the expression levels of such genes.^7^ A series of hypoxia-related prognostic signatures that attempt to correlate the hypoxia-regulated gene expressions to the clinical outcomes of patients with breast cancer have been proposed.^3^^,^^6^^,^^8^ Alternatively, hypoxia-related proteins, such as hypoxia-inducible factor-1α and its downstream targets, can be assessed using immunohistochemistry on tissue sections. The presence and localization of these proteins can indicate the extent of hypoxia in the tumor. Both approaches require separate assays and techniques, which may involve additional sample processing and costs outside the current breast cancer pathways and might be unavailable in some settings because of resource limitations. Hematoxylin and eosin (H&E)–stained tissue slides, on the other hand, are part of standard clinical practice and readily available in pathology laboratories. H&E-stained slides provide rich morphologic information, capturing cellular architecture, tissue organization, and tumor-stromal interactions. With the increasing adoption of whole slide imaging (WSI) scanners, H&E-stained slides routinely prepared for pathologic examination are now being digitized and stored for artificial intelligence (AI)–assisted analysis. Recent studies have shown the capabilities of deep learning (DL) models to predict gene expression, somatic mutations, or recurrence scores from H&E-stained WSIs of breast histology, showing potential to produce biologic insights and novel biomarkers.^9^^,^^10^

This study demonstrates a novel application of weakly supervised DL to detect hypoxia patterns in breast cancer tissue from H&E-stained histology only. Contrary to fully supervised approaches, weakly supervised ones do not necessarily require tissue- or cell-level manual annotations.

More specifically, this article shows how a weakly supervised DL model (HypOxNet), which was trained only with weak sample-level labels corresponding to hypoxia-regulated gene expression, was able to evaluate hypoxia in the context of the tumor histomorphology.

## Materials and Methods

**Figure 1:**
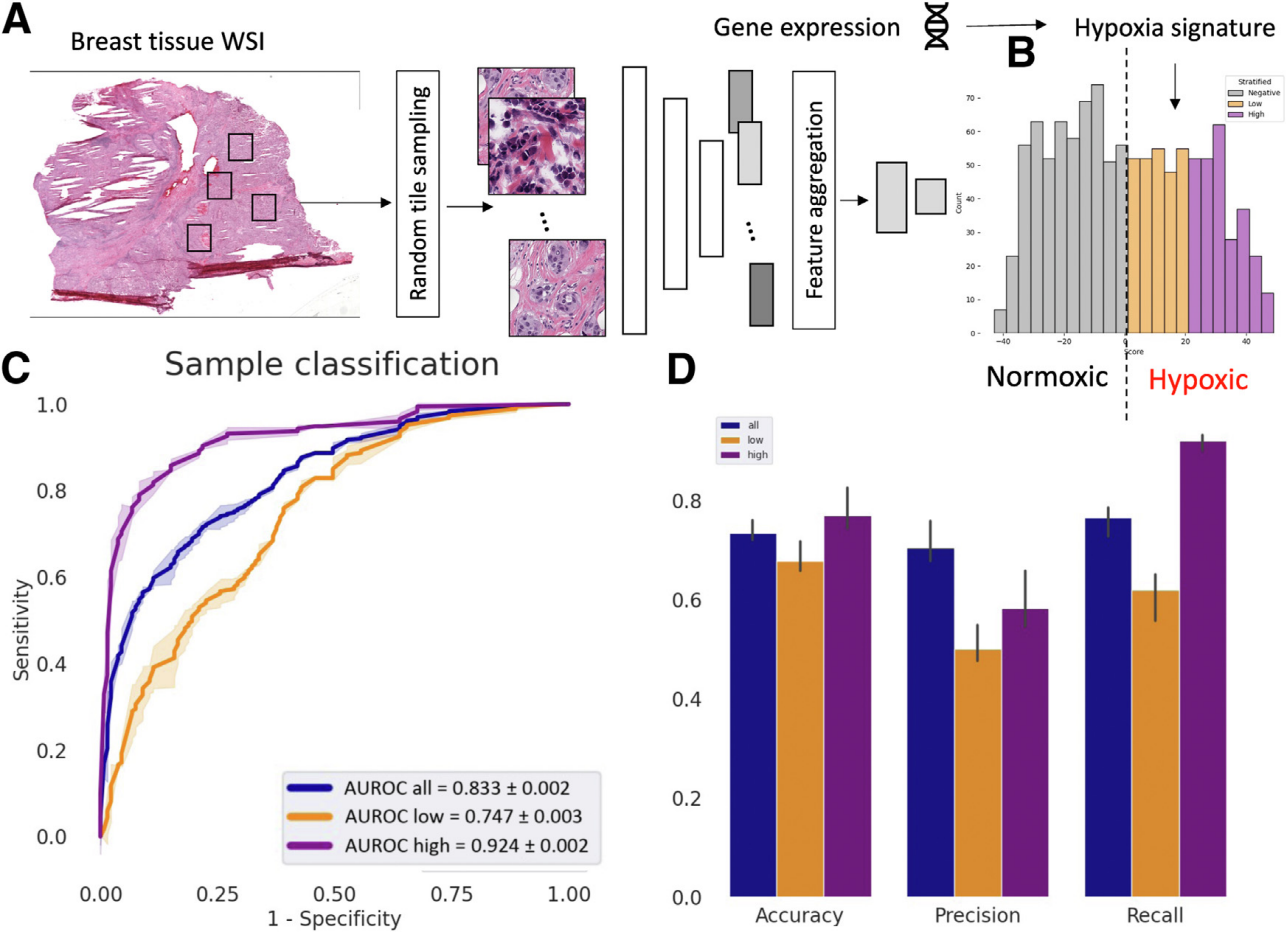
Hypoxia prediction neural network (HypOxNet). **A:** Approach. Whole slide images (WSIs) of breast biopsies from The Cancer Genome Atlas database^11^ are first divided into small tiles. Bags of tiles are passed through a multiple instance learning convolutional neural network with an attention pooling layer. The network is trained to classify the bags of tiles as hypoxic or normoxic, according to their hypoxia signature.^12^**B:** Hypoxia score distribution.^4^ Samples with a positive score were considered as hypoxic, whereas the remaining ones were considered as normoxic. **C:** Average areas under the receiver operating characteristic curve (AUROCs) on the left-out test set for all (Buffa score >0), low (20 > Buffa score > 0), and high (Buffa score >20). **D:** Additional performance metrics on the test sets. Performance on the training sets is shown in Supplemental Figure S2. *n* = 253 (with 3 random train-test splits; **C**). Original magnification, ×40 (**A**).

The HypOxNet model was trained to classify WSIs of H&E-stained breast tumor tissues (×40 magnification) into hypoxic or normoxic (Figure 1). Data from The Cancer Genome Atlas,^11^ which contains both WSIs of primary site tumors and a wide range of corresponding gene expressions measured by RNA sequencing, were used in this study (*https://portal.gdc.cancer.gov*, last accessed December 9, 2024).

Ground truth annotations were obtained through a stratification process based on the Hypoxia Buffa signature^12^ scores using the hypoxia-regulated gene expressions determined from RNA-sequencing data available in The Cancer Genome Atlas database. Details on these hypoxia score calculations can be found in the study by Bhandari et al.^4^

The Hypoxia Buffa signature was used to measure the hypoxia scores in The Cancer Genome Atlas breast cancer cohort (*n* = 1016), ranging from −43 to 47 (Figure 1B). The samples were further stratified into hypoxic and normoxic, based on the value of these hypoxia scores (Figure 1B). This binary stratification, based on the hypoxia score, served as the weak labels associated with each WSI.

### WSI Preprocessing

Each WSI was divided into smaller tiles of size 256 × 256 pixels at ×40 magnification to manage the large image sizes and focus on local tissue structures. Next, tiles with <50% tissue content were discarded to ensure that only relevant tissue regions were used for training the model. For this purpose, first, each of the RGB (red, green, blue) tiles was converted to grayscale. The background is typically lighter in H&E-stained images (closer to white), whereas tissue regions are darker because of the stain. The Otsu^13^ thresholding method was chosen to segment tissue from the background. Here, pixels below the Otsu^13^ threshold value were considered part of the tissue. Finally, the proportion of pixels identified as tissue versus the total pixels in the tile was calculated and used for training and testing only the RGB tiles containing >50% tissue.

To enhance model robustness and generalization, during training, these tiles were subject to on-the-fly geometrical augmentation (random rotations and random flips) as well as spectral augmentation (random hue modification, random gamma corrections, and random noise).

### Network Architecture and Training Details

The selected RGB tiles were further used to train a multiple instance learning convolutional neural network model to predict sample-level labels (Figure 1A). More specifically, the HypOxNet model was trained to classify “bags” of tissue tiles (*T*_*k*_^*i*^) with *i* = 1,…,*N* corresponding to each sample *k* with sample-level binary labels (*L*_*k*_) derived from the hypoxia hallmark signature (Buffa).^4^ The convolutional layers of the model were initialized with weights from a VGG-19 model^14^ pretrained on the ImageNet data set.^15^ The convolutional feature vectors corresponding to each tile *conv*(*T*_*k*_^*i*^) of the model were first reduced to 512 features each using Global Average Pooling (GAP) and then aggregated into a single bag-level feature vector using a pooling mechanism followed by a classification layer.

(1) $$L_{k}=\text{softmax}\left(ReLu\left(Pool\left(F_{k}^{1},F_{k}^{2},\ldots ,F_{k}^{N}\right)\;\cdot W_{1}+b_{1}\right)\right)$$

where *N* is the number of input image patches, F_k_^i^ = GAP(*conv*(*T*_*k*_^*i*^), W_1_, b_1_ are the corresponding weights of the dense layer. Two different pooling strategies for multiple instance learning feature aggregation were evaluated: Max-Pool and Attention Pool.^16^ During training, 20 image tiles were randomly selected per sample for each iteration. A stochastic gradient descent with a learning rate of 0.0003 and a cross-entropy loss function to optimize the model weights during 100 epochs was used. At inference, all the image tiles containing tissue from each test sample unseen during training were passed through the HypOxNet models. The models were generated using TensorFlow in Python 3.6 (*https://www.python.org/downloads/release/python-360*).

### Morphology Tile Analysis

The model predictions in terms of histomorphology and image features were investigated further. For a trained HypOxNet model, randomly sampled 2000 image tiles were selected (10 patches per sample) from 100 normoxic and 100 hypoxic samples from the test sets. Next, each single patch was passed through the trained model (bags of one instance), and those with scores of <0.9 were discarded. For each of the patches classified as normoxic or hypoxic with high confidence (a score of >0.9; *n* = 576), the gray-level co-occurrence matrix–based texture features^17^ implemented in Python's scikit-image were calculated (*https://scikit-image.org*, last accessed December 9, 2024):

(2) $$\text{Homogeneity}=\sum_{i}^{N}\;\sum_{j}^{N}\frac{P\left(i,j\right)}{1+{\left(i-j\right)}^{2}}$$

(3) $$\text{Energy}=\sqrt{\sum_{i}^{N}\sum_{j}^{N}{P\left(i,j\right)}^{2}}$$

(4) $$\text{Correlation}=\sum_{i}^{N}\;\sum_{j}^{N}\frac{\left(i-\mu_{i}\right)\left(j-\mu_{j}\right)}{\sqrt{\sigma_{i}^{2}\sigma_{j}^{2}}}$$

where *N* denotes the number of gray levels (256), and *P*(*i*,*j*) is the grayscale normalized value at position *i* and *j* of the patch. Textural features were generated for each tile converted to grayscale with distance at 1, rotation at 0, 45, 90, and 135 degrees, and then averaged (*https://scikit-image.org/docs/0.7.0/api/skimage.feature.texture.html#skimage.feature.texture.greycomatrix*, last accessed December 9, 2024).

### Cell Morphology Analysis

The model predictions in terms of cell morphology were investigated next. This analysis used the tiles from breast tissues from the MoNuSaC challenge data set,^18^ which contains patches with cell-level contour annotations for various types of cells, including epithelial cells and macrophages. Similar to the previous experiment, a previously trained HypOxNet model was used to classify the MoNuSaC tiles as normoxic or hypoxic and discarded those with classification scores of <0.9. For each of the patches classified as normoxic or hypoxic with high confidence, the corresponding manual annotations available in the data set were used to compute the morphologic descriptors for both epithelial and macrophage cells. More precisely, the classic binary shape descriptors (area, eccentricity, extent, diameter, perimeter, and solidity) were extracted using the scikit-image toolkit.

(5) $$\text{Eccentricity}=\sqrt{1-\frac{b^{2}}{a^{2}}}$$

where *b* = minor axis length, and *a* = major axis length of a connected component binary mask. An elongated object would have an eccentricity value close to 1, whereas a round object would have an eccentricity value close to 0.

(6) $$\text{Circularity}=\frac{4\pi \cdot \mathrm{Area}}{{\mathrm{Perimeter}}^{2}}$$

A perfect circle would have a circularity value of 1, whereas the value goes down as far as 0 for highly noncircular shapes.

(7) $$\text{Extent}=\frac{A_{object}}{A_{BBox}}$$

where $A_{object}$ represents the area of the object (in pixels), whereas $A_{BBox}$ represents the area of its bounding box.

## Results

### Multiple Instance Learning Detects Breast Cancer Hypoxia in H&E-Stained WSIs

The weakly supervised DL HypOxNet models were trained and validated to predict hypoxia status with image patches of tissue from 530 normoxic samples (hypoxia score, <0) and 486 hypoxic samples (hypoxia score, >0) from The Cancer Genome Atlas database.^11^ The data set was randomly split into training (3/4) and test (1/4) sets. A threefold cross-validation yielded an average area under the curve of 0.833 ± 0.002 (Figure 1C) on the test set. The HypOxNet models performed better at detecting hypoxia in samples with a high Buffa signature score (>20) than those with a score between 0 and 20 (area under the receiver operating characteristic curve, 0.92 versus 0.75) (Figure 1C). Figure 1D shows the average accuracy, precision, and recall scores on the left-out test sets. These results were obtained with a Max-Pool feature aggregation strategy. Comparative results with Attention Pooling are available in Supplemental Figure S1. Performance on the training sets is shown in Supplemental Figure S2. Results on a different hypoxia signature (MSigDb^3^) are available in Supplemental Figure S3.

### Texture Analysis Reveals Differences in Hypoxic Tissues

**Figure 2:**
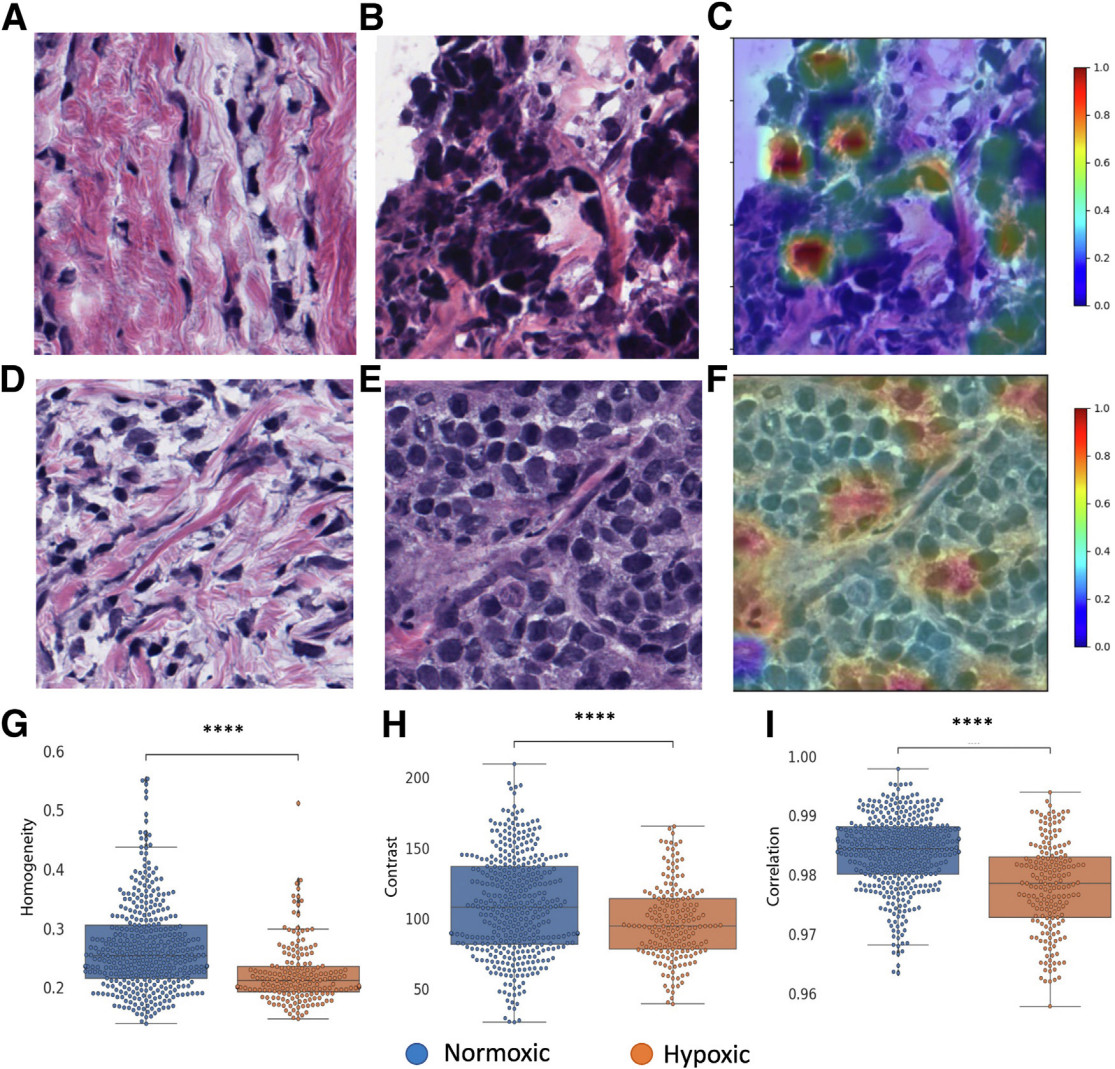
Tile-level texture analysis. **A** and **D:** Examples of tiles classified by HypOxNet as normoxic. **B** and **E:** Examples of tiles classified by HypOxNet as hypoxic. **C** and **F:** Corresponding class activation maps overlayed on the hypoxic tiles. **G**–**I:** Gray-level co-occurrence matrix–based texture feature box plots of individual tiles (size = 512 × 512) classified as hypoxic (orange) and normoxic (blue). Additional texture feature comparison can be found in Supplemental Figure S4. *n* = 576 (**G**–**I**). ∗∗∗∗*P* < 0.0001. Original magnification, ×40 (**A**–**F**).

Figure 2, A and D, shows examples of tiles classified as normoxic by the model, whereas Figure 2, B and E, shows examples of tiles classified as hypoxic. Figure 2, C and F, shows the tile regions highlighted as important for the classification (blue to red) by the class activation maps.

The homogeneity (Figure 2G), energy (Figure 2H), and correlation (Figure 2I) texture feature average values were significantly lower when calculated on the hypoxic tissue tiles than on those classified by the HypOxNet models as normoxic. Additional texture feature comparison can be found in Supplemental Figure S4.

### Shape Analysis Reveals Differences in Epithelial and Macrophage Cells from Hypoxic Tissue

**Figure 3:**
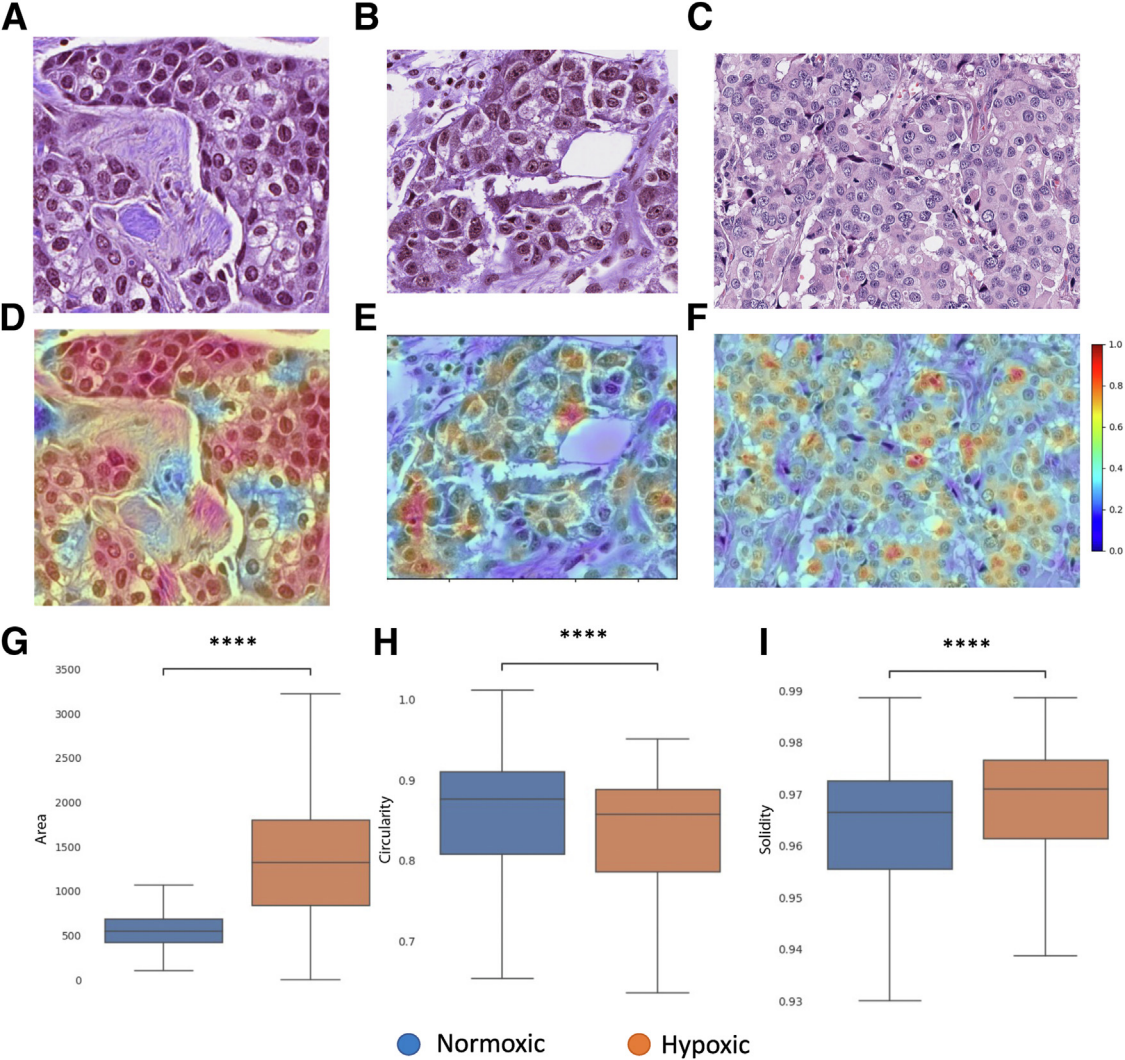
Cell-level shape analysis of epithelial cells from the MoNuSaC annotated data set.^18^**A:** Example of tiles containing epithelial cells classified by HypOxNet as normoxic. **B** and **C:** Examples of tiles classified by HypOxNet as hypoxic. **D**–**F:** Corresponding class activation maps overlayed on the tiles. **G**–**I:** Box plots of binary shape descriptors of epithelial cells in tiles classified as normoxic (blue) and hypoxic (orange) by the deep learning model. Shape descriptors were computed using the implementation available (*https://scikit-image.org/docs/stable/api/skimage.measure.html#skimage.measure.regionprops*, last accessed December 9, 2024). Additional shape feature comparison can be found in Supplemental Figure S5. *n* = 2560 (**G**–**I**). ∗∗∗∗*P* < 0.0001. Original magnification, ×40 (**A**–**F**).

**Figure 4:**
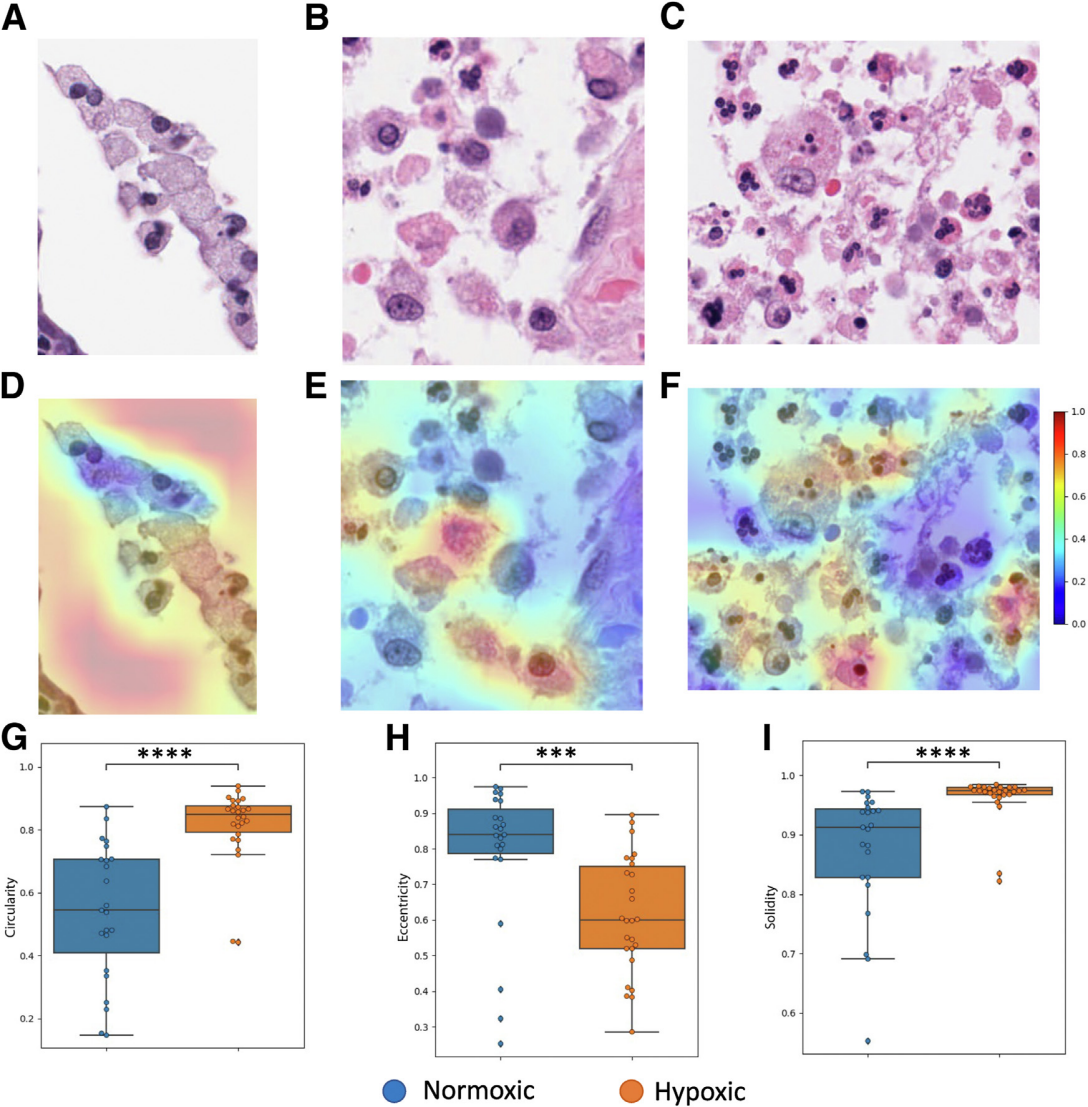
Cell-level shape analysis of macrophae cells from the MoNuSaC annotated data set.^18^**A:** Example of tiles containing macrophages classified by HypOxNet as normoxic. **B** and **C:** Examples of tiles classified by HypOxNet as hypoxic. **D**–**F:** Corresponding class activation maps overlayed on the tiles. **G**–**I:** Box plots of binary shape descriptors of epithelial cells in tiles classified as normoxic (blue) and hypoxic (orange) by the deep learning model. Shape descriptors were computed using the implementation available (*https://scikit-image.org/docs/stable/api/skimage.measure.html#skimage.measure.regionprops*, last accessed December 9, 2024). Additional shape feature comparison can be found in Supplemental Figure S6. *n* = 96 (**G**–**I**). ∗∗∗*P* < 0.001, ∗∗∗∗*P* < 0.0001. Original magnification, ×40 (**A**–**F**).

An example of a tile containing epithelial cells classified as normoxic is shown in Figure 3A, whereas Figure 3, B and C, shows tiles classified as hypoxic. Figure 3, D–F, shows the tile regions highlighted as important for the classification (blue to red) by the class activation maps. The morphologic analysis shows that epithelial cells belonging to hypoxic regions have, on average, higher area (Figure 3G) and solidity (Figure 3H) and a lower circularity (Figure 3I). An example of a tile containing macrophage cells classified as normoxic is shown in Figure 4A, whereas tiles classified as hypoxic are shown in Figure 4, B and C. Figure 4, D–F, shows the tile regions highlighted as important for the classification (blue to red) by the class activation maps. The shape analysis suggests that macrophage cells belonging to regions classified as hypoxic by the multiple instance learning model have, on average, a higher circularity (Figure 4G) and solidity (Figure 4H) and a lower eccentricity (Figure 4I). Additional shape feature comparison can be found in Supplemental Figures S5 and S6.

## Discussion

This study demonstrates that a weakly supervised DL model can distinguish hypoxia in the context of the tumor's histopathologic characteristics from routine H&E-stained slides. More specifically, it shows that H&E-stained slides contain histomorphology biomarkers of hypoxia.

Gene expression profiling and hypoxia scores typically require separate assays and techniques, which may involve additional sample processing and costs. AI analysis of H&E-stained slides, on the other hand, uses existing pathology slides, eliminating the need for additional experiments. H&E-stained slides are part of standard clinical practice and are readily available in pathology laboratories. Such a DL hypoxia detection model could potentially be easily integrated into the pathology workflow, which would streamline the process and facilitate clinical adoption. Moreover, gene expression profiling or advanced molecular analyses may be less accessible in resource-constrained settings. AI-based analysis of H&E-stained slides could be a more feasible alternative in such situations. Linking AI-detected hypoxia patterns in H&E-stained slides with clinical outcomes could potentially help establish prognostic or predictive significance, enabling faster patient stratification and treatment decision-making.

Preliminary analysis suggests that both epithelial cells and macrophages in hypoxic regions have significant morphologic differences compared with ones belonging to nonhypoxic regions. Tumor-associated macrophages play a crucial role in the tumor microenvironment and have a significant impact on tumor development. Hypoxia induces several changes in tumor-associated macrophages that promote tumor progression, such as immunosuppression, angiogenesis, or extracellular matrix remodeling.^19^^,^^20^

As a proof of principle, this study showed that HypOxNet can be successfully applied to breast cancer. However, this approach could potentially be applied to other tumor types as well, where hypoxia is an important factor in treatment selection.^21^, ^22^, ^23^

## Disclosure Statement

None declared.

## Supplemental Data

Supplemental Figure S1 Comparative study of HypOxNet feature aggregation strategies. **A:** Average areas under the receiver operating characteristic curve on the left-out test set. **B:** Additional performance metrics on the test sets. *n* = 253 (with 3 random train-test splits; **A**). AUC, area under the curve.

Supplemental Figure S2 Performance of the HypOxNet models on the training set. **A:** Average areas under the receiver operating characteristic curve on the training sets (three random train-test splits). **B:** Additional performance metrics on the training sets. AUC, area under the curve.

Supplemental Figure S3 HypOxNet model trained and tested on the hypoxia hallmark for the MSigDb signature^3^ on The Cancer Genome Atlas whole slide image. **A:** Average area under the receiver operating characteristic curve on the left-out test set. **B:** Additional performance metrics on the test sets. *n* = 80 (with 3 random train-test splits; **A**). AUC, area under the curve.

Supplemental Figure S4 Tile-level texture analysis. Additional gray-level co-occurrence matrix–based texture feature box plots of individual tiles (size = 512 × 512) classified as hypoxic (orange) and normoxic (blue). *n* = 576. ∗∗∗∗*P* < 0.0001. ASM, angular second-moment; ns, nonsignificant.

Supplemental Figure S5 Cell-level shape analysis of epithelial cells from the MoNuSaC annotated data set.^15^ Box plots of additional binary shape descriptors of epithelial cells in tiles classified as normoxic (blue) and hypoxic (orange) by HypOxNet. Shape descriptors were computed using the implementation available *(**https://scikit-image.org/docs/stable/api/skimage.measure.html#skimage.measure.regionprops*, last accessed December 9, 2024). *n* = 2560. ∗∗∗∗*P* < 0.0001. ns, nonsignificant.

Supplemental Figure S6 Cell-level shape analysis of macrophages from the MoNuSaC annotated data set.^15^ Box plots of additional binary shape descriptors of macrophage cells in tiles classified as normoxic (blue) and hypoxic (orange) by HypOxNet. Shape descriptors were computed using the implementation available (*https://scikit-image.org/docs/stable/api/skimage.measure.html#skimage.measure.regionprops*, last accessed December 9, 2024). *n* = 96. ∗*P* < 0.05, ∗∗∗∗*P* < 0.0001. ns, nonsignificant.
