# Supplementary figures and images for: Computational Pathology Detection of Hypoxia-Induced Morphologic Changes in Breast Cancer
Source: Am J Pathol. 2024 Dec 26;195(4):663–70. doi: 10.1016/j.ajpath.2024.10.023 (PMC12179535; doi:10.1016/j.ajpath.2024.10.023)

**A**

## Sample Classification

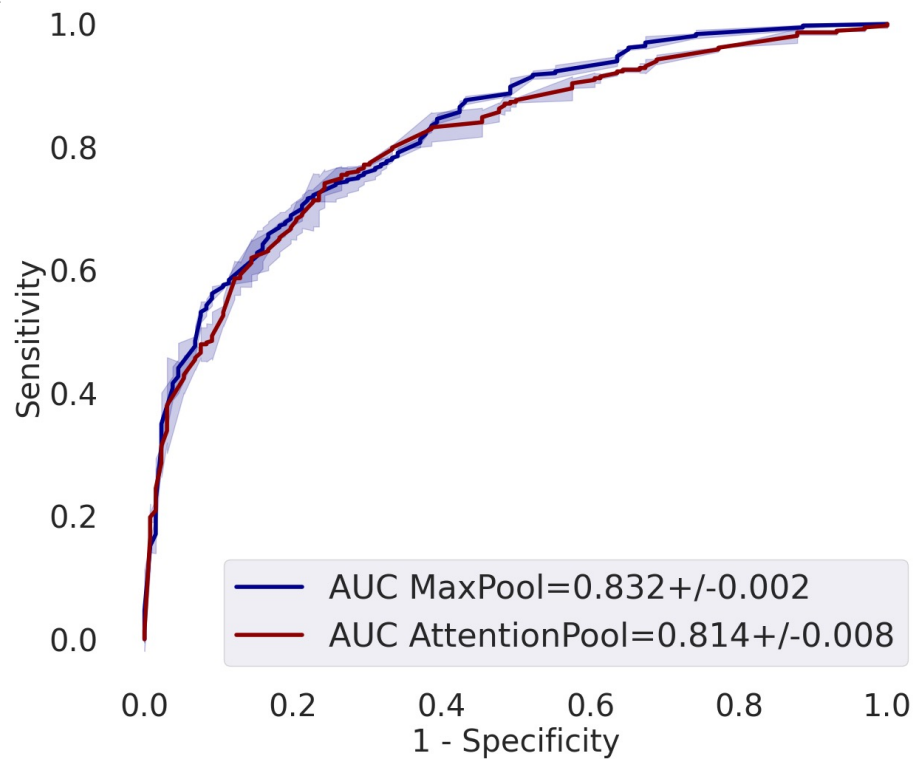**B**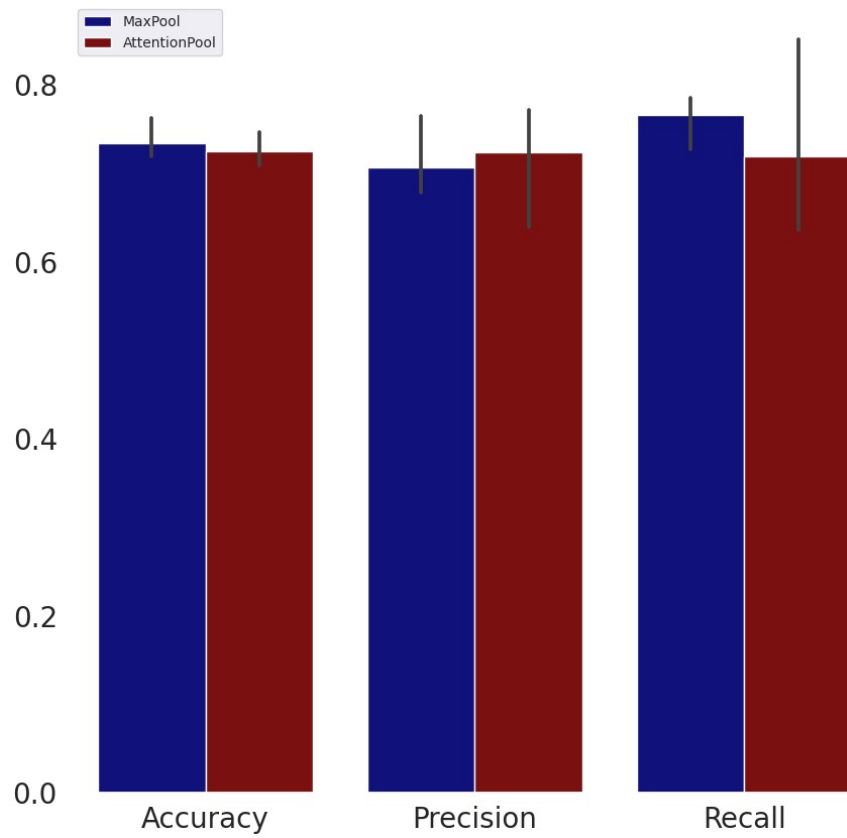

Supplement: Supplemental Figure S1 — Comparative study of HypOxNet feature aggregation strategies. A: Average areas under the receiver operating characteristic curve on the left-out test set. B: Additional performance metrics on the test sets. n = 253 (with 3 random train-test splits; A). AUC, area under the curve. [file mmc1.pdf]

**A**

### Sample Classification

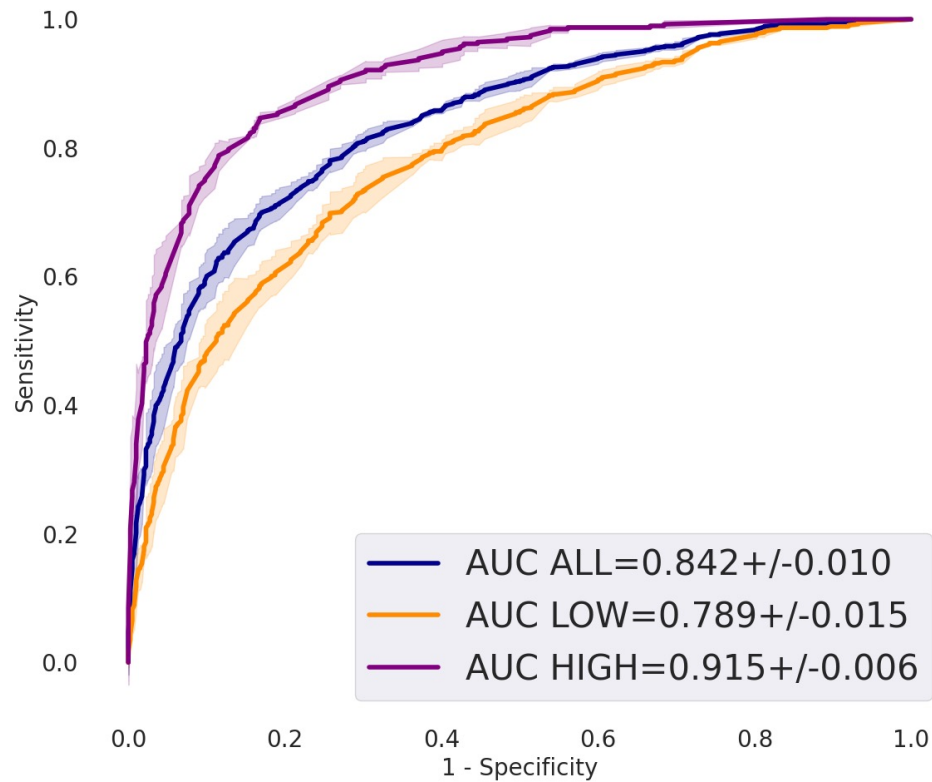**B**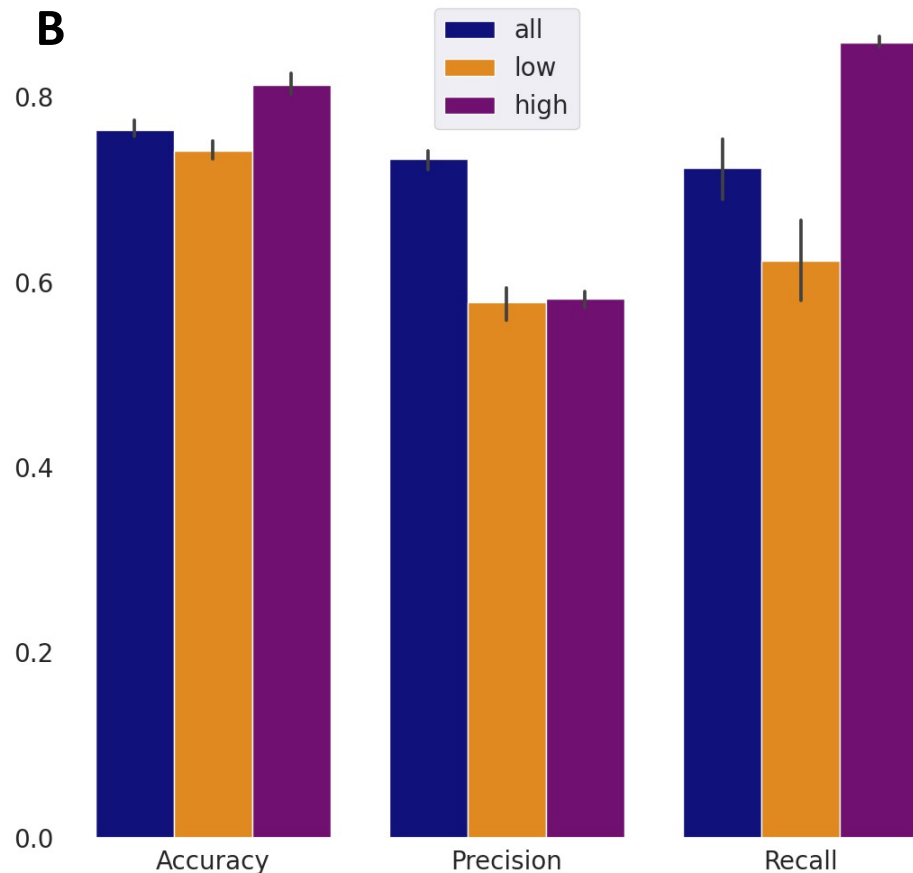

Supplement: Supplemental Figure S2 — Performance of the HypOxNet models on the training set. A: Average areas under the receiver operating characteristic curve on the training sets (three random train-test splits). B: Additional performance metrics on the training sets. AUC, area under the curve. [file mmc2.pdf]

**A**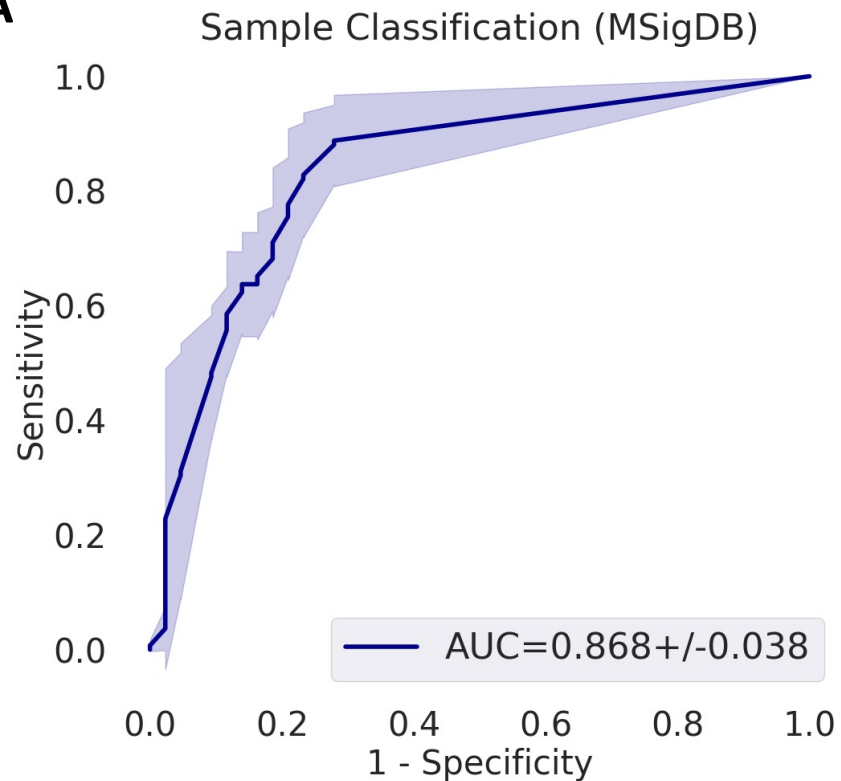**B**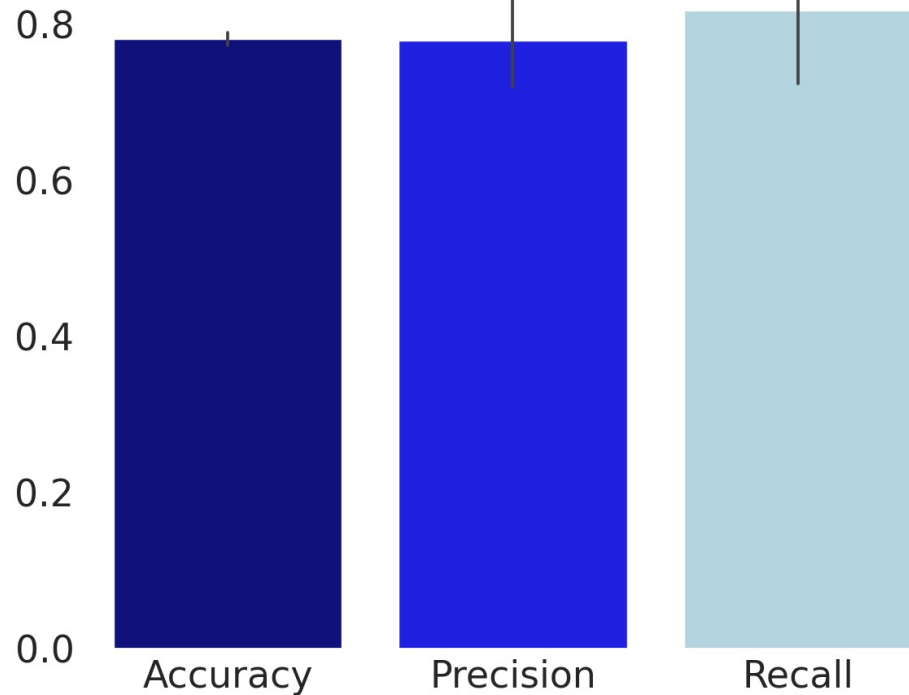

Supplement: Supplemental Figure S3 — HypOxNet model trained and tested on the hypoxia hallmark for the MSigDb signature3 on The Cancer Genome Atlas whole slide image. A: Average area under the receiver operating characteristic curve on the left-out test set. B: Additional performance metrics on the test sets. n = 80 (with 3 random train-test splits; A). AUC, area under the curve. [file mmc3.pdf]

dissimilarity

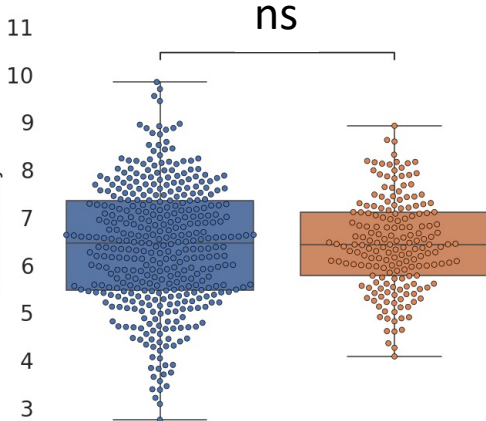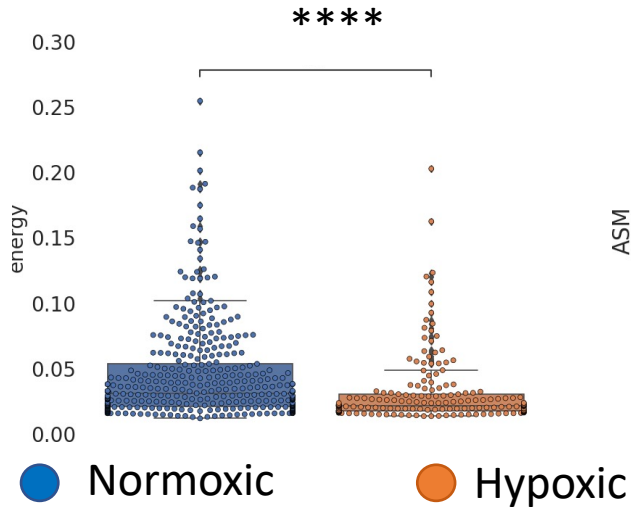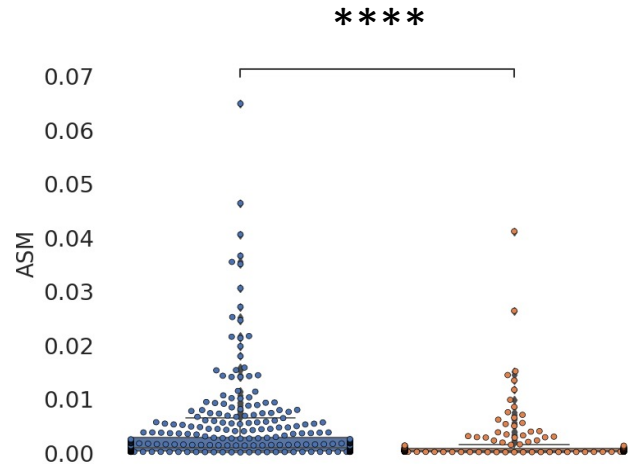

Supplement: Supplemental Figure S4 — Tile-level texture analysis. Additional gray-level co-occurrence matrix–based texture feature box plots of individual tiles (size = 512 × 512) classified as hypoxic (orange) and normoxic (blue). n = 576. ∗∗∗∗P < 0.0001. ASM, angular second-moment; ns, nonsignificant. [file mmc4.pdf]

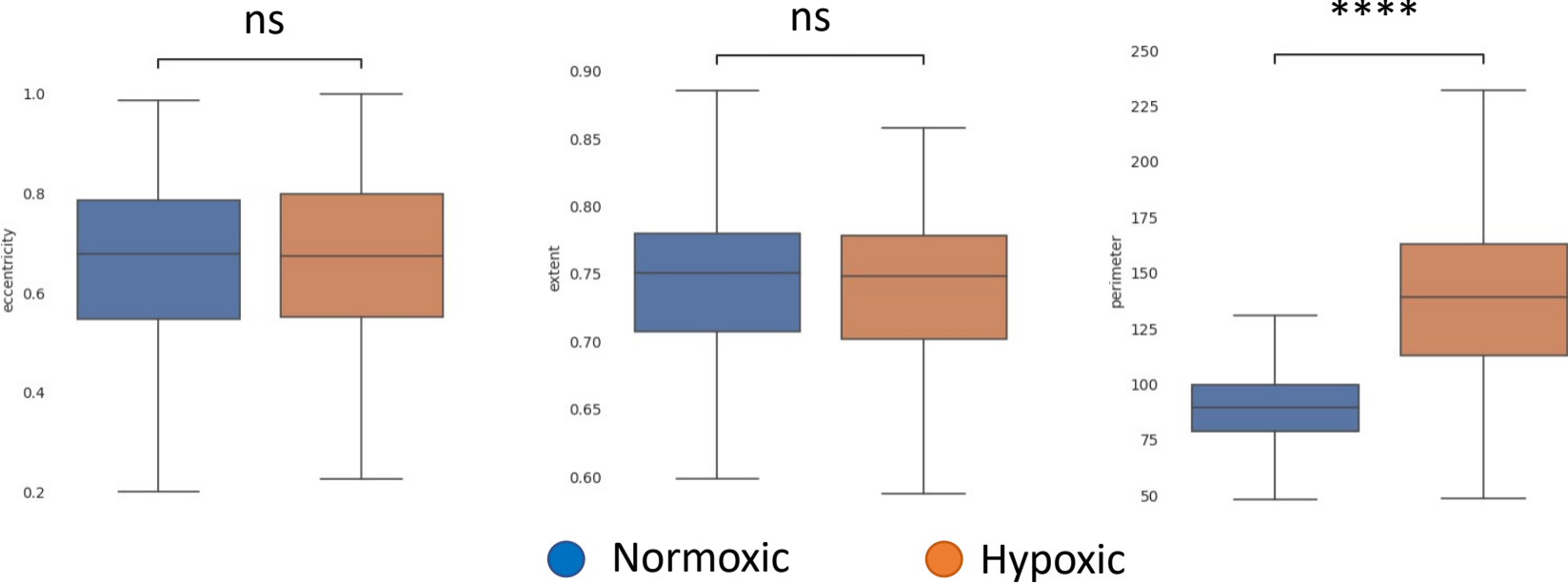

Supplement: Supplemental Figure S5 — Cell-level shape analysis of epithelial cells from the MoNuSaC annotated data set.15 Box plots of additional binary shape descriptors of epithelial cells in tiles classified as normoxic (blue) and hypoxic (orange) by HypOxNet. Shape descriptors were computed using the implementation available (https://scikit-image.org/docs/stable/api/skimage.measure.html#skimage.measure.regionprops, last accessed December 9, 2024). n = 2560. ∗∗∗∗P < 0.0001. ns, nonsignificant. [file mmc5.pdf]

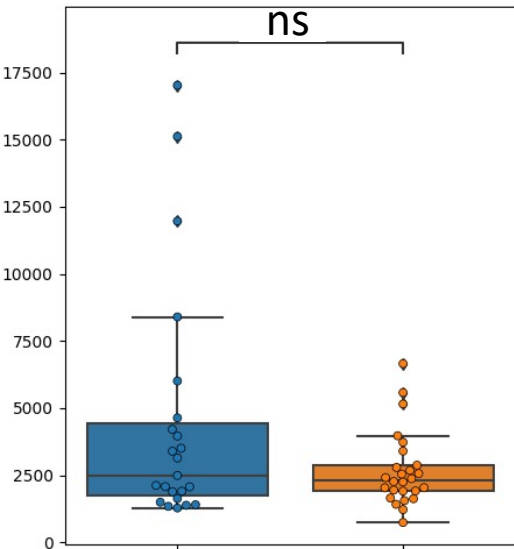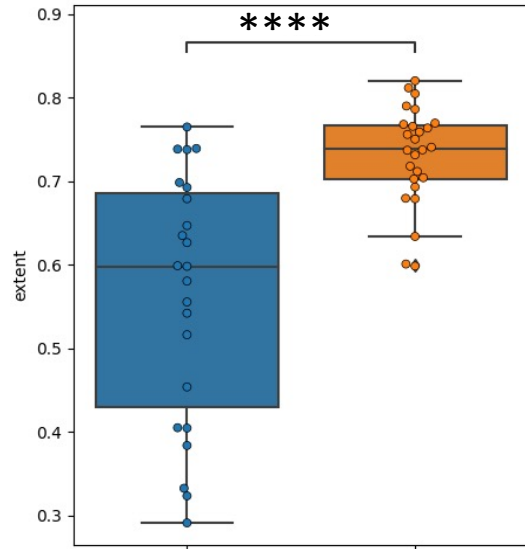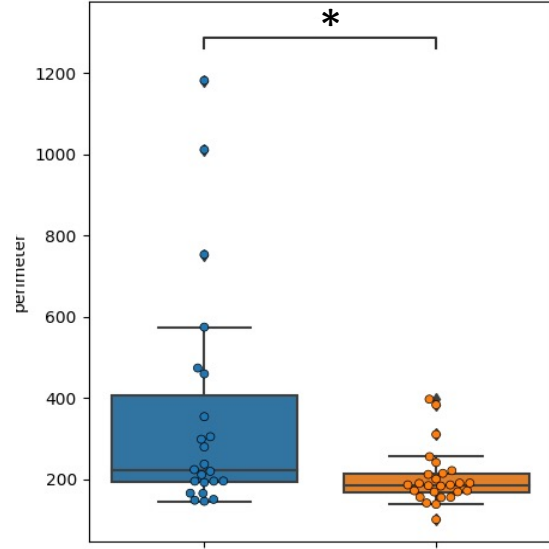

● Normoxic

● Hypoxic

Supplement: Supplemental Figure S6 — Cell-level shape analysis of macrophages from the MoNuSaC annotated data set.15 Box plots of additional binary shape descriptors of macrophage cells in tiles classified as normoxic (blue) and hypoxic (orange) by HypOxNet. Shape descriptors were computed using the implementation available (https://scikit-image.org/docs/stable/api/skimage.measure.html#skimage.measure.regionprops, last accessed December 9, 2024). n = 96. ∗P < 0.05, ∗∗∗∗P < 0.0001. ns, nonsignificant. [file mmc6.pdf]
